# Dopant-stimulated growth of GaN nanotube-like nanostructures on Si(111) by molecular beam epitaxy

**DOI:** 10.3762/bjnano.9.17

**Published:** 2018-01-15

**Authors:** Alexey D Bolshakov, Alexey M Mozharov, Georgiy A Sapunov, Igor V Shtrom, Nickolay V Sibirev, Vladimir V Fedorov, Evgeniy V Ubyivovk, Maria Tchernycheva, George E Cirlin, Ivan S Mukhin

**Affiliations:** 1 St. Petersburg Academic University, Khlopina 8/3, 194021 St. Petersburg, Russia; 2 ITMO University, Kronverkskij 49, 197101 St. Petersburg, Russia; 3 Ioffe Institute, Politekhnicheskaya 29, 194021 St. Petersburg, Russia; 4 St. Petersburg State University, 7/9 Universitetskaya emb., 199034 St. Petersburg, Russia; 5 Peter the Great St. Petersburg Polytechnic University, Polytechnicheskaya 29, 195251 St. Petersburg, Russia,; 6 Institut d’Electronique Fondamentale, UMR 8622 CNRS, University Paris Sud, University Paris-Saclay, 91405 Orsay cedex, France

**Keywords:** A3B5 on Si, epitaxy, GaN, MBE, nanowires, nanotubes, nanotube-like nanostructures, Si

## Abstract

In this paper we study growth of quasi-one-dimensional GaN nanowires (NWs) and nanotube (NT)-like nanostructures on Si(111) substrates covered with a thin AlN layer grown by means of plasma-assisted molecular beam epitaxy. In the first part of our study we investigate the influence of the growth parameters on the geometrical properties of the GaN NW arrays. First, we find that the annealing procedure carried out prior to deposition of the AlN buffer affects the elongation rate and the surface density of the wires. It has been experimentally demonstrated that the NW elongation rate and the surface density drastically depend on the substrate growth temperature, where 800 °C corresponds to the maximum elongation rate of the NWs. In the second part of the study, we introduce a new dopant-stimulated method for GaN nanotube-like nanostructure synthesis using a high-intensity Si flux. Transmission electron microscopy was used to investigate the morphological features of the GaN nanostructures. The synthesized structures have a hexagonal cross-section and possess high crystal quality. We propose a theoretical model of the novel nanostructure formation which includes the role of the dopant Si. Some of the Si-doped samples were studied with the photoluminescence (PL) technique. The analysis of the PL spectra shows that the highest value of donor concentration in the nanostructures exceeds 5∙10^19^ cm^−3^.

## Introduction

Gallium nitride quasi-one-dimensional nanostructures such as nanowires (NWs) and nanotubes (NTs) synthesized by means of plasma-assisted molecular beam epitaxy are attracting a lot of interest due to their prospective use as basic elements for new generation optoelectronic devices [1–2]. The most important properties of these structures in terms of potential device applications are high crystal quality and efficient light emission [3–4]. It has been previously demonstrated that solid Ga(In, Al)N alloys can be used in the production of light-emitting and light-absorbing devices covering wide spectral range [5–6]. One of the main NW features is their small footprint and large surface-to-volume ratio, which allows the growth of these defect-free structures on highly mismatched substrates, e.g., GaN NWs on Si [7–8]. NWs usually possess high crystal quality due to the effective mechanical stress relaxation at a distance of about one NW base diameter from the substrate. Nanowires synthesized on Si are very promising nanostructures in the field of photovoltaics. In case of silicon-based solar cells (SCs), reflection can be reduced from 30% (pure Si) down to 3% without deposition of multilayer antireflection coatings or involvement of complicated postgrowth techniques generally used for modification of the SC surface roughness [9]. The fabrication of a simple SC based on GaN NWs on Si(111) can be obtained via proper NW doping and formation of a p–n junction at Si substrate–GaN NW interface. Recently it has been theoretically demonstrated that optimization of the doping level and NW array morphology can lead to a power conversion efficiency of over 20% in such a simple SC [9]. The development of controllable methods of GaN nanostructure growth and doping on Si substrates opens up new possibilities for integration of III–V materials with established CMOS technology. The latter issue represents one of the bottlenecks of modern opto-nanoelectronics [10].

The discovery of carbon NTs in 1991 unfolded new possibilities for the development of future generation nanoelectronic devices. Growing interest in the tubular structures motivated the synthesis of different semiconductor NTs, one of them being GaN NTs. Comparing to NWs and nanocolumns, NTs offer a new degree of freedom due to possible confinement effects. It has been previously demonstrated that GaN NTs may be synthesized using the following methods: 1) chemical vapor deposition of nitrogen precursor with gallium precursor in the presence of catalysts such as Ni, In or Au [11–13]; 2) formation of a GaN shell over the NW template (e.g., ZnO) followed by the template NW removal [14]; 3) selective area molecular beam epitaxy (MBE) growth of GaN on sapphire (111) substrates over titanium mask [15]; and 4) MBE deposition of GaN on Si(111) substrates covered by a silicon oxide layer in the absence of a doping flux [16].

Compared to other widely studied III–V NWs (e.g., Al(Ga, In)As), which can be synthesized by MBE on Si substrates via a vapor–liquid–solid (VLS) mechanism that uses catalyst droplets, GaN NWs grow according to the self-induced mechanism in the absence of catalyst [17–18]. In general, this mechanism does not require surface preparation. The self-induced NWs grow when specific growth conditions are satisfied and the material nucleates easier on the top of a NW than on its sidewalls [19], which are usually nitrogen-rich (N-rich) conditions. In this case, a N-rich reconstruction should be on the NW top polar facet [20] enabling good bonding of Ga atoms, while on nonpolar sidewalls, the adsorbed atoms are weakly bonded and diffuse to the top facet or can be desorbed. Self-induced formation of NWs has been discovered on Al_2_O_3_ [21] and was successfully reproduced on other substrates [22]. The most promising in terms of potential device application substrates is Si(111) due to cost reasons. The deposition of an AlN buffer layer is usually carried out prior to growth of GaN to maintain vertical nature of the NWs. Selective area growth is also a possibility. It was demonstrated that growth of GaN NWs starts with the formation of a nanoisland, followed by a geometrical transition when the nanoisland footprint reaches a specific size [23].

The development of optoelectronic devices based on GaN nanostructures requires precise control of the type and level of doping. The growth, formation process and doping of GaN NWs on Si(111) substrates have been extensively studied during the last decade [24–26]. It has been experimentally demonstrated that Si and Mg doping (materials typically used to obtain n- and p-type conductivity) strongly affects the growth kinetics and thus the NW morphology, leading to lateral broadening of the growing structures [27]. In particular, the presence of Si atoms leads to an increased probability of atomic step formation on the nonpolar sidewalls of the NW, and as a result, to its radial extension during growth [27]. It has been experimentally demonstrated that in the presence of a sufficient Si doping flux, a radial gradient of the dopant concentration exists inside the n-GaN NW and a less doped core accompanied by thin heavily doped shell may form [28–29]. A similar NW doping effect has been obtained in the growth study of InN NWs [30]. It should be noted that the dopant concentration in the heavily doped shell may significantly exceed the solubility limit of Si atoms in planar GaN layers (which is about 5∙10^19^ cm^−3^ [31]). In the case of 2D layers, such a high doping level leads to an increase of the elastic stress and massive emergence of threading dislocations [32–34] followed by transition to three-dimensional growth of nanoislands [33,35]. The phenomenon of the Si solubility limit elevation in GaN NWs is usually explained again through effective stress relaxation due to the large surface area of these nanostructures.

This work is dedicated to the study of Si-doped GaN NWs and NT-like structure synthesis on Si(111) substrates by means of plasma-assisted molecular beam epitaxy in N-rich conditions. We investigated the impact of the silicon oxide layer, substrate temperature and gallium flux on the NW formation. To the best of our knowledge, in this paper we demonstrate for the first time self-organized dopant-activated growth of high crystal quality GaN NT-like nanostructures on Si(111) substrates covered with a thin AlN buffer layer created using the MBE technique. The synthesized nanostructures were studied with photoluminescence (PL), scanning electron microscopy (SEM) and transmission electron microscopy (TEM) techniques. We demonstrate high crystal quality and the possibility of heavy Si doping of the GaN structures. In the end, we present a theoretical approach explaining the NT-like nanostructure formation.

## Results and Discussion

### Growth technique

In our experiments, we used p-type Si(111) substrates that were treated with the Shiraki cleaning procedure prior to loading into the MBE chamber, where each substrate was annealed in ultrahigh vacuum. The annealing temperature varied from sample to sample in the range of 850 to 1000 °C. We then cooled down the substrate to 650 °C for deposition of a few nanometer thick AlN layer. Then the substrate was heated to the growth temperature and GaN was deposited. All experiments were carried out in a Veeco GENIII MBE machine. A Riber RF valved plasma source was used to provide the atomic nitrogen flux.

### Nanowire formation

**Figure 1:**
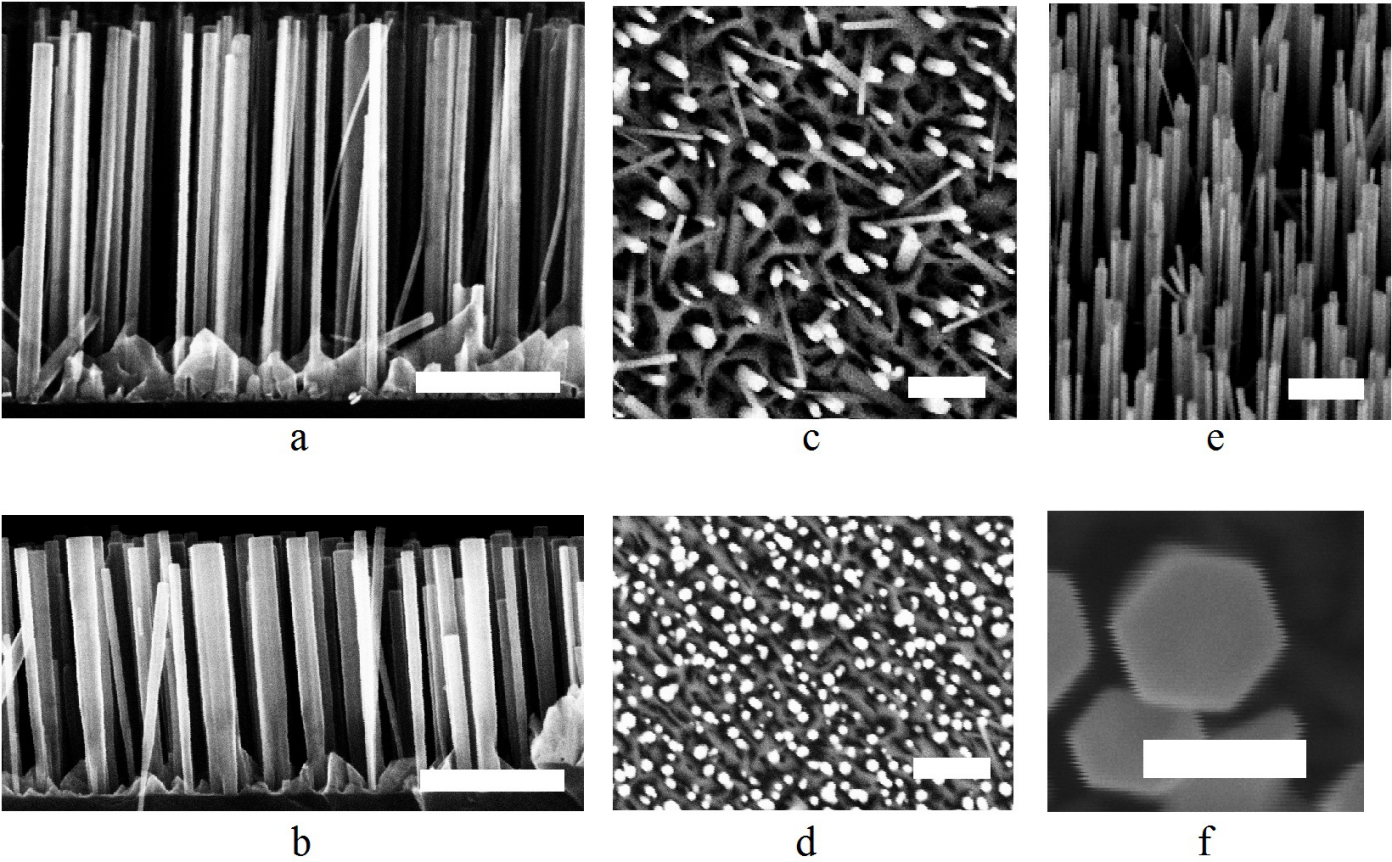
Scanning electron microscope (SEM) images of the nanowire (NW) arrays: a) image of the array grown on the substrate that underwent low temperature annealing (850 °C, sample 2); b) images of the array grown on the substrate that underwent high temperature annealing (1000 °C, sample 7); c) top view image of the sample 2; d) top view image of the sample 7 (clearly indicating a higher surface density compared to sample 2); e) tilted view of the sample 2; f) enlarged view on the cross-section of a single NW. The scale bar in all images is 400 nm, except for (f) where it is 100 nm.

The analysis of the SEM images (Figure 1a–f) and experimental data allows us to make several important conclusions. First, the cross-section of the synthesized NWs is hexagonal (see Figure 1f). Second, we registered the highest NW elongation rate value of 35.9 nm/h on a substrate that underwent low-temperature (850 °C) annealing. It should be mentioned that in this case a silicon oxide layer covering the substrate was removed only partially or was not removed at all. This conclusion is based on the analysis of in situ reflection high-energy electron diffraction (RHEED) patterns: we did not observe (7 × 7) a Si surface reconstruction pattern while cooling down the substrate that was subjected to the low temperature annealing. On the contrary, when the high temperature annealing (at least 920 °С) was applied, we observed a clear (7 × 7) reconstruction, implying that all the oxide was desorbed. Surprisingly, in the latter case, the highest elongation rate value was only 18.6 nm/h, which is twice slower than in the presence of oxide. At the same time, the NW surface density increased threefold (see Figure 1). It is well known that a major role in the nucleation and growth of NWs is played by growth species diffusion. We could thus conclude that Ga adatom kinetics on the substrate strongly depend on the surface layer chemistry and can be altered by the annealing procedure.

We have also considered the volume of incorporated material in our experimental data analysis using following formula: *M* = *V*_gr_σ((*D* + *d*)/2)^2^ where *V*_gr_ is the average elongation rate, σ is the surface density of the nanostructure, *D* is the average top diameter of the nanostructure and *d* is the average base diameter of the nanostructure. No sufficient change in material incorporation was observed between samples grown for about 30 h but subject to different temperature annealing procedures, though both the elongation rate and surface density were affected, as mentioned previously.

Another aim of our study was to analyze the influence of the substrate temperature on the growth process. We varied the substrate temperature over a narrow range of 790–820 °C. It turned out that the NW elongation rate and surface density critically depend on the temperature. The optimum temperature value corresponding to the highest observed NW elongation rate was 800 °C. According to the experimental data, reducing the temperature by just ten degrees to 790 °C leads to a NW elongation rate decrease of 30%. At the same time, their surface density significantly increases, indicating a reduction of the adatom mean free path limited by the incorporation in a crystal lattice and consequently by faster nucleation, though the material consumption is not notably affected. On the other hand, when the substrate temperature was 810 °С we observed a 60% reduction of material incorporation with a corresponding decrease in the elongation rate and surface density. An increase of the substrate temperature by 10 °C leads to a 97% decrease of the material consumption. The observed phenomenon is intuitively understandable: a temperature increase leads to the reduction of the adatom mean free path limited by desorption that results in slower crystallization.

We have also carried out a growth experiment with an elevated flux of Ga. Experimental data allows us to conclude that, in this case, no sufficient change in material consumption occurs, although an increase of the Ga flux leads to an increase of the NW surface density with a reduction in the elongation rate or, in other words, stimulation of 2D growth.

### Nanotube-like nanostructure formation

**Figure 2:**
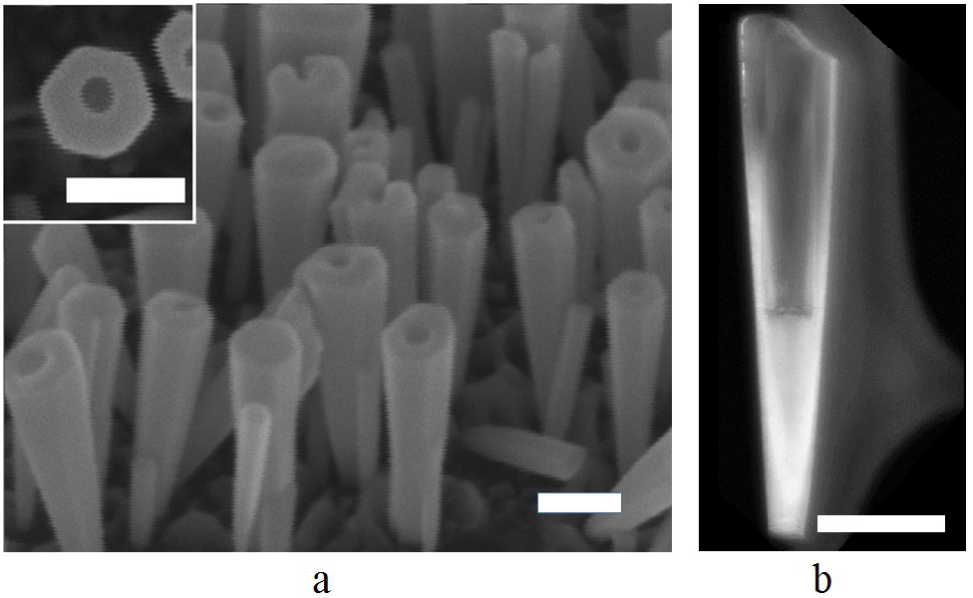
SEM (a) and TEM (b) images of the synthesized nanotubes. The scale bar is 100 nm.

In early works it was reported that Si doping leads to changes in the GaN NW morphology [27]. It was demonstrated that the higher the flux, the stronger tapering occurs. In our work, a lateral extension of the NWs was not observed for temperatures of the Si doping cell below 1060 °C. To obtain a high doping of the NWs we synthesized a sample on the substrate that underwent the high temperature annealing by increasing the Si cell temperature to 1160 °C. SEM images of the grown sample (Figure 2a) show the anticipated effect of the NW lateral extension towards the top facet and a slight reduction of the elongation rate. Surprisingly, the analysis of the top-view SEM images revealed that the synthesized nanostructures not only significantly widen but also a geometrical cavity settles along their axis: instead of NWs GaN NT-like structures were grown. As can be seen from the images (see Figure 2a), the nanostructures have a regular hexagonal shape and a preferential growth direction perpendicular to the substrate surface. In the TEM image (Figure 2b) a hollow cavity with nearly vertical walls can be seen inside the nanostructure. The cavity occupies about half of the nanostructure length and has a horizontal bottom. A bright shade of a thin carbon layer supporting the nanostructure during the measurement can be seen to the right of the nanostructure.

### Optical properties of silicon-doped GaN nanostructures

**Figure 3:**
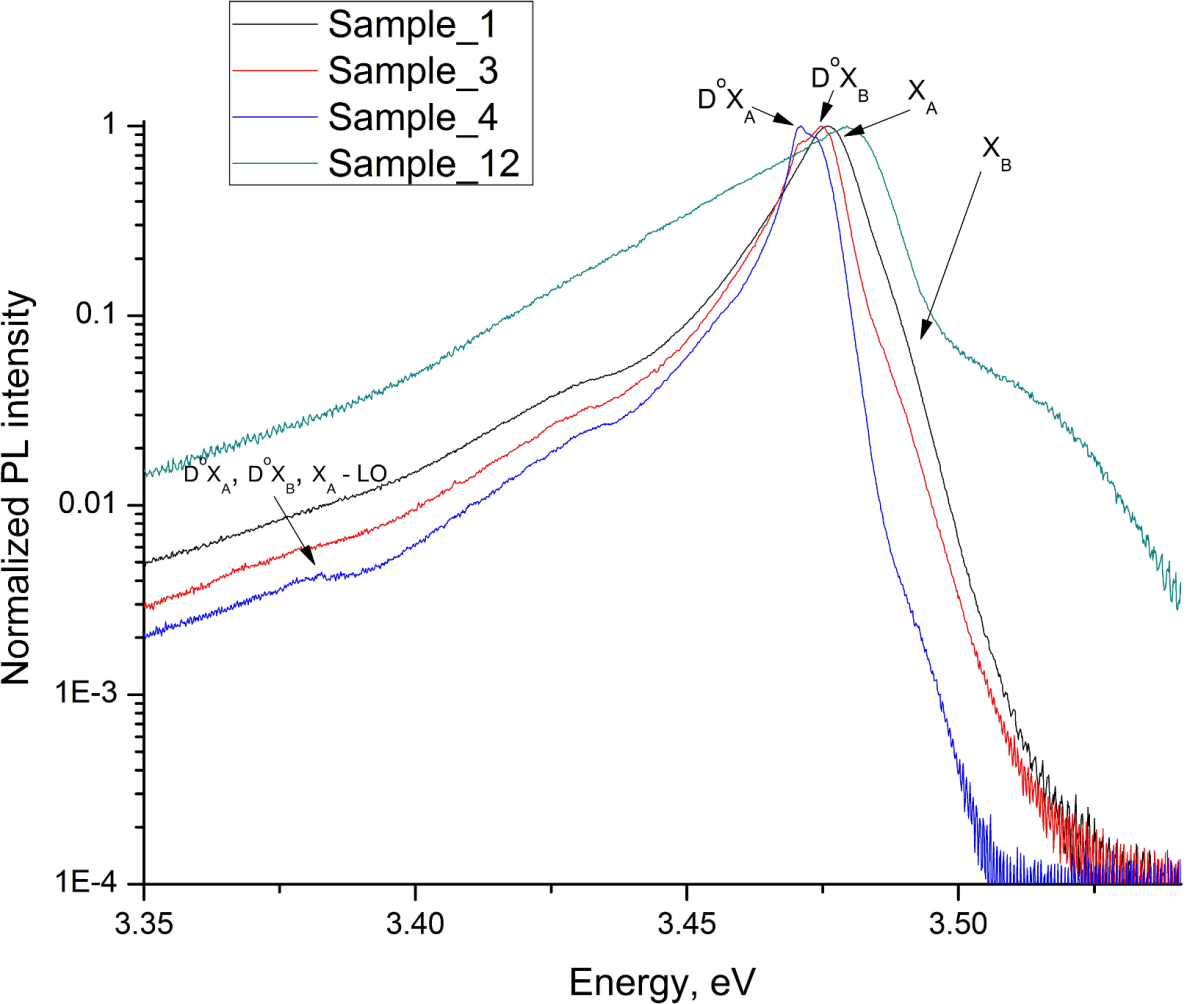
Photoluminescence (PL) spectra of the synthesized GaN nanostructures.

In this work, we study the influence of the Si doping on the photoluminescence (PL) spectra of the synthesized structures. In the Table 1 one can find a description of the studied samples. Figure 3 shows the PL spectra from samples with various doping levels (samples 1, 3, 4 and 12). The spectra were obtained at a temperature of 10 K by using a helium closed cycle cryostat. PL was excited using a HeCd laser with a wavelength of 325 nm and detected using a PMI Hamamatsu R298 detector. The power density of the laser excitation was 10 W/cm^2^.

**Table 1:** Parameters of the samples analyzed with photoluminescence. The 2D doping level is the doping level for planar GaAs grown with a corresponding growth rate and Si doping flux.

Sample	Si effusion cell temperature	Diameter, nm	2D doping level, cm^−3^

sample 1	undoped	73	–
sample 3	1050 °C	79	3·10^17^ cm^−3^
sample 4	1060 °C	153	4·10^17^ cm^−3^
sample 5	1100 °C	81	3·10^18^ cm^−3^
sample 12	1160 °C	87	3·10^19^ cm^−3^

The analysis of the PL spectra shows that no yellow luminescence occurs in our samples, indicating high crystal quality of the synthesized GaN nanostructure arrays. It is well known that if the wave vector *k* of an incident wave is orthogonal to the *c*-axis of the NW crystal lattice, then three PL lines corresponding to free excitons of light X_A_, heavy X_B_ and crystal field split X_C_ holes are allowed as well as two PL lines corresponding to D^0^X_A_ and D^0^X_B_ excitons bound to neutral donors [36]. In our study we did not expect acceptor–bound excitons since the studied nanostructures were Si-doped.

Figure 3 shows that four exciton lines of the PL spectra of our samples can be distinguished, namely: X_A_, X_B_, D^0^X_A_ and D^0^X_B_. As one can see, in our spectra, phonon replicas of exciton transitions related to a longitudinal optical phonon with 93 meV energy [37] (X_A_ – 1 LO, D^0^X_A_ – 1 LO and D^0^X_B_ – 1 LO) are observed. Features in the region of 3.43 eV indicate transitions related to structural defects [38].

The spectrum of sample 1 (black curve) contains a high-intensity X_A_ peak and a weak D^0^X_B_ peak which is a manifestation of the background Si doping of the GaN NWs and always appears with an impurity concentration of around 10^17^ cm^−3^ [39–40]. The spectrum of sample 1 presents a dominant peak corresponding to the appearance of free X_B_ excitons. The latter fact demonstrates that when the Si doping flux is lower than 1∙10^−10^ Torr (corresponding to a Si cell temperature lower than 1100 °C), the doping level is determined by the background doping. As can be seen in Figure 3, donor bound excitons are much stronger in doped NWs from samples 3 and 4 (red and blue curves, correspondingly), however the free exciton PL band X_A_ is also present.

The difference in the ratios of D^0^X_A_ to D^0^X_B_ peak intensities for samples 3 and 4, which were synthesized under very similar growth conditions (difference in the doping cell temperature is only 10 °C and growth time is about 30 h for sample 3 while for sample 4 it is 92 h), could be related to the difference in the NW diameters for those samples together with a strong surface recombination found in the NWs. We should note that the location of all three exciton transition peaks is the same for samples 1, 3 and 4, which means that their doping level does not exceed 10^18^ cm^−3^ [37].

The position of the PL spectrum maximum of sample 5 is sufficiently red shifted. According to [37] such an effect is an indication of the impurity band formation with a corresponding carrier concentration at the level of 3∙10^18^ cm^−3^.

The PL spectrum of sample 12, which was grown under the highest doping flux in our experiment series, significantly differs from the PL spectra of other considered samples 1, 3, and 4. The position of the PL maximum is shifted towards higher energy and the PL band is significantly broader. A typical form of the emission spectrum shows that at high doping level impurity levels, a band merges with the conduction band [37]. In the case of bulk GaN, such a phenomenon can be observed when the doping level reaches its limit – 5∙10^19^ cm^−3^. Thus, the approximate doping level for planar layers (see Table 1) is in close proximity with the doping level estimated via PL data analysis. So we conclude that the low-temperature PL is an effective noncontact method of assessing the doping level in GaN NWs.

### Theoretical model

According to modern theoretical approaches, axial growth of GaN NWs at high growth temperature can be explained by the near zero nucleation barrier on the top polar facet of this wurtzite structure, in comparison to its nonpolar sidewalls [27,41]. Crystallization on the NW top facet starts earlier and reduces the Ga adatom concentration on this facet. In N-rich conditions, the nucleation barrier on the top facet was lower than on the sidewalls since there is a huge excess of nitrogen on the NW top [19]. A difference between the adatom chemical potential (related to concentration) on the sidewalls and on the top facet results in a diffusion flux [42].

**Figure 4:**
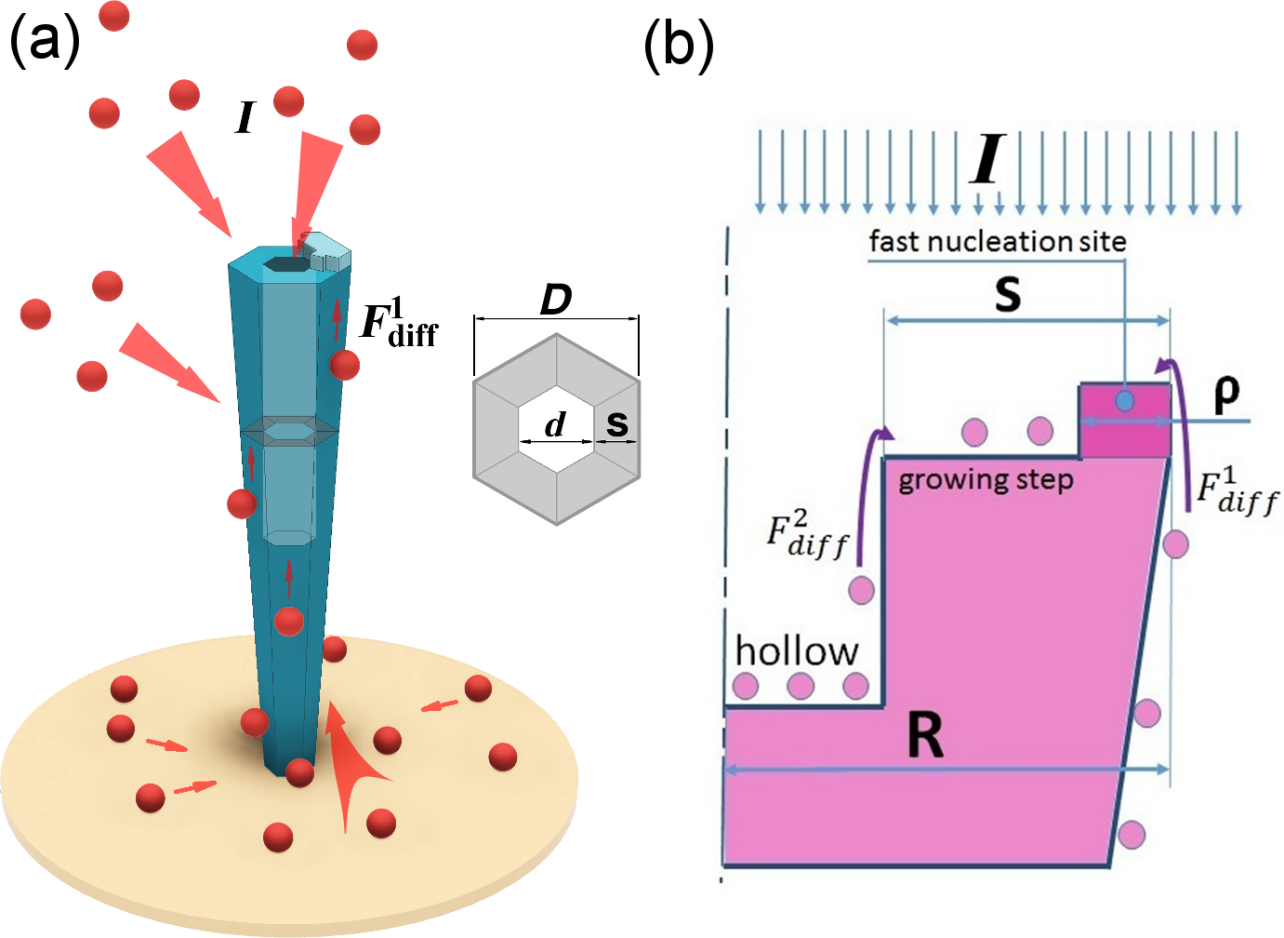
a) Nanotube growth schematics and b) model nanotube cross-section.

In the absence of Si, a concentration of Ga adatoms on the sidewalls is not sufficient for GaN nucleation and no radial extension occurs. The growth dynamics changes when Si flux is applied: it has been demonstrated [27] that a high concentration of Si doping lowers the GaN nucleation barrier on the NW sidewalls and lateral growth occurs. From the other point-of-view, a very high concentration of Si doping in the case of N-rich GaN planar growth leads to Si surface segregation [31]. When the segregation occurs, the concentration of Si atoms at the crystal surface can be an order of magnitude higher than in the bulk [43]. A similar effect of doping level elevation (which value may even exceed the Si solubility limit in bulk GaN) was observed in the surface layer of GaN NWs [27–28]. The preferential incorporation of Si in the outer part (shell) of the NW leads to formation of point defects, which reduce the nucleation barrier along the NW top facet boundary. Besides, the incoming flux on the outer part of the top facet is higher than in the central part, since in addition to direct impingement flux, a diffusion flux from the NW sidewalls is also present (Figure 4).

Summarizing the results from above, the formation of GaN NTs can be explained assuming that a highly Si-doped shell formation leads to a zero nucleation barrier along the NW top facet boundary so that Ga adatoms, which have diffused to the facet from the sidewalls, cannot reach its central area due to a very fast incorporation along the boundary. In other words, at some critical Si concentration, the mechanism of NW growth changes from nucleation-mediated to transport-mediated.

As can be seen in Figure 2, NTs do not grow directly on the substrate surface. Instead, first a NW is formed and then a NW to NT growth mode transition occurs. We assume that this effect is related to a variation in the radial distribution of the Si dopants along the NW axis. In the lower part of the NW, elastic stress should reduce the Si incorporation barrier in the NW lattice, especially in the central part where the stress is higher, therefore, the central part of the NW base is silicon-enriched. Further away from the substrate, the strain rapidly reduces, and the lattice constant in the NW is reduced to that of bulk GaN. Here Si atoms preferentially join the outer part of the NW [44]. So we conclude that the Si dopant concentration in the outer part of the NW is increasing with the NW height, and when its value reaches a certain limit, corresponding to a zero nucleation barrier, the NT growth mode occurs.

Transmission electron microscopy image (Figure 2b) shows that thickness of the NT increases towards the top, while the hollow diameter does not change. We suppose that lattice planes confine the hollow part due to surface energy minimization and thus determine the NT inner diameter, *d*.

The considerations stated above allow us to develop a theoretical model of NT formation. We assume that a fast nucleation process along the NW top facet boundary governs the axial growth rate d*L*/d*t*. When the nucleation site (ring-shaped island) along the boundary is formed, it starts to grow laterally and the formation of an atomic layer proceeds until its edge reaches the lattice plane confining the hollow part.

We consider that the NT-like nanostructure is cylindrically symmetric in our model, so the top facet is a ring of a width *s* and the fast nucleation site is a ring of a width ρ, which is about the maximal distance that an adatom can diffuse from the sidewalls towards the facet center before incorporation (Figure 4b). The incoming flux consists of two parts: direct impingement 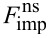 and diffusion flux from the sidewalls 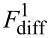. The following is the material balance equation for the nucleation ring:

[1]
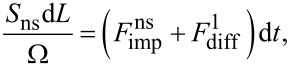


where *S*_ns_ = π*D*ρ(1 − ρ/*D*) is the area of the nucleation site, Ω is the volume per GaN pair. The diffusion length of Ga is relatively small since we did not observe a length over diameter decreasing dependency typical for diffusion-limited growth [45]. So another diffusion flux could be written in the following form: 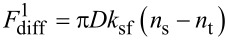 where *D* is the diameter of the top facet, *k*_sf_ is the velocity of diffusion transition, *n*_s_ is the adatom concentration on the sidewalls near the top and *n*_t_ is the adatom concentration on the top facet. Here we assume that the adatom concentration on the NW top is nearly zero. A direct impingement flux could be easily estimated in this case as a product of the ring area and deposition flux density, *F*_imp_ = *S*_ns_*I*, where *I* is the gallium flux (species/nm^2^s). Putting all expressions above together, we arrive at:

[2]
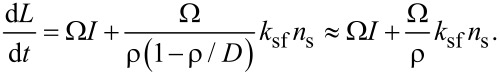


From another point of view, the lateral growth of a new monolayer on the top facet relates to direct impingement 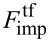 and diffusion flux from the sidewalls and from the hollow part (
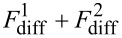
). Therefore, another material balance equation may be written as

[3]
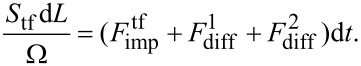


Here *S*_tf_ is the top facet area. The geometrical properties that lead to multiple scattering and the desorption of adatoms from the inner walls are neglected and we consider that all atoms deposited in the hollow part diffuse to the NT top facet: 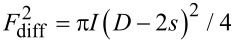. Consequently, it follows from Equation 3:

[4]
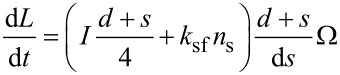


Putting together Equation 2 and Equation 4 we get:

[5]
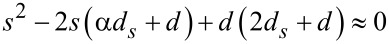


Here, α = d/ρ – 1, *d*_s_ = 2*k*_sf_*n*_s_ / *I*. The adatom concentration on the NW sidewalls near the top is determined by the impingement on the NW sidewalls, diffusion flux from the substrate and desorption. It can be expressed in the following way [46]: *n*_s_ = *n*_s_ (*z*) = *n*_0_ + *n*_1_ exp(−*z*/λ), where *n*_0_ and *n*_1_ are constants, *z* is the distance from substrate, λ is the adatom diffusion length on the sidewalls. Consequently, the parameter *d*_s_ = *d*_s_ (*z*) depends on *z* too. Putting the last expression into Equation 5, we obtain a NT wall thickness dependence on distance *z* as:

[6]
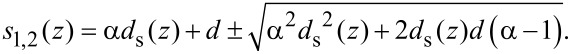


The analysis of the obtained formulas allows us to conclude that an increase of the distance *z* leads to an increase of the NT wall thickness – the same phenomena observed in our experiments. Effectively, diffusion flux reduces with time due to the elongation of the NTs, which leads to a reduction of the elongation rate of the structure and its radial extension.

## Conclusion

To conclude, we investigated the influence of the growth parameters on the formation and morphological properties of the synthesized arrays of quasi-one-dimensional GaN nanostructures on Si(111) substrates covered with a thin AlN buffer layer. We demonstrated that the annealing procedure affects the nanostructure growth rate and surface density: the elongation rate on a substrate that underwent the low temperature annealing is twice as high as for the substrate that underwent the high temperature oxide removal procedure and reached 35.9 nm/h. At the same time, the surface density is lower in the first case.

The elongation rate was found to significantly change when the substrate temperature varied in a narrow 20 °C range. We obtained an optimum substrate temperature of 800 °C, corresponding to the highest elongation rate. The lower temperature leads to an increase of the surface density and decrease of the elongation rate, while higher temperatures lead to fast a reduction of both the elongation rate and density.

We demonstrated, for the first time, that a high intensity Si doping flux can activate the growth of GaN NT-like nanostructures on Si(111) substrates covered with a thin AlN buffer layer. Analysis of the SEM and TEM images of the grown NTs show their high crystallinity. Additionally, we present a theoretical model explaining the obtained phenomena.

Doped GaN nanostructures were investigated using the PL technique. We observed a broadening of the spectrum for the sample grown with the Si effusion cell temperature above 1160 °C, which indicates a merge of the impurity band with conduction band, which in turn indicates that the doping level of these structures reached 5·10^19^ cm^−3^.
